# Two new species of freshwater crab of the genus *Aparapotamon* Dai & Chen, 1985 (Crustacea, Brachyura, Potamidae) from Yunnan, China

**DOI:** 10.3897/zookeys.1056.63755

**Published:** 2021-08-20

**Authors:** Qi-Hong Tan, Xiao-Juan Zhou, Jie-Xin Zou

**Affiliations:** 1 Research Laboratory of Freshwater Crustacean Decapoda & Paragonimus, School of Basic Medical Sciences, Nanchang University, 461 Bayi Avenue, Nanchang City, Jiangxi Province 330006, China Nanchang University Nanchang China; 2 Key laboratory of Poyang Lake Environment and Resource Utilization, Ministry of Education, Nanchang University, 1299 Xuefu Avenue, Nanchang City, Jiangxi Province 330031, China Nanchang University Nanchang China

**Keywords:** *
Aparapotamon
*, freshwater crab, new species, taxonomy, 16S rRNA

## Abstract

Two new species of freshwater crab of the genus Potamid *Aparapotamon* Dai & Chen, 1985 are described from Yunnan Province, southwest China. Morphological comparisons were made between the two new species and type materials of other 11 species of *Aparapotamon*. *Aparapotamonbinchuanense***sp. nov.** and *A.huizeense***sp. nov.** can be separated from their congeners by the shape of the epibranchial tooth, the frontal view of the cephalothorax, the male first gonopod, and the female vulvae. The molecular analyses based on partial mitochondrial 16S rRNA gene are also included. This study brings the number of *Aparapotamon* species to 13.

## Introduction

Crabs of the family Potamidae Ortmann, 1896 (Crustacea, Decapod, Brachyura) spend their whole life history in freshwater or terrestrial environments (Yeo et al. 2008). The juvenile crabs hatch directly from large and yolky eggs, and there is no larval phase in their life history, so they are considered true freshwater crabs (Yeo et al. 2008; Daniels et al. 2015). Due to their low fecundity and poor dispersal abilities, these crabs are easily blocked by geographical barriers, and their phylogeny often closely reflects relevant historical geological events (Shih et al. 2009, 2011; Fang et al. 2015; Ji et al. 2016; Jia et al. 2018).

Previous studies have shown that China has the world’s highest number of freshwater crab species (Dai 1999; Cumberlidge et al. 2011; Shih and Ng 2011; Daniels et al. 2015; Chu et at. 2018a). Despite this substantial diversity, the rate of discovery remains high (Chu et al. 2018b; Huang et al. 2018a, b, 2020a, b; Naruse et al. 2018; Zou et al. 2018; Gao et al. 2019; Wang et al. 2019a, b, 2020a, b; Mao and Huang 2020). With the two new species described in this study, Yunnan has a total of 18 genera and 67 species, highest among all provinces in China (Chu et al. 2018a; Wang et al. 2020). Yunnan is a possible center of origin for the family Potamidae and is located in the southwest of China (Shih and Ng 2011). It is at the junction of the Asiatic Plate and the Indian Plate (Harrison et al. 1992), the geological movement remains active, the complex geographical features of this area have contributed to the rapid differentiation of crabs (Shih et al. 2009), so species of this area is richer than that in other places at the same latitude such as Guangxi Zhuang Autonomous Region and Guangdong Province (Shih and Ng 2011).

*Aparapotamon* was established by Dai and Chen (1985), and eleven species have been reported so far. Since all the species are from Yunnan, Sichuan, Guangxi, Hunan, Hubei, Chongqing, and Shaanxi but with only *A.gracilipedum* Chen & Chang, 1982 known from Henan Province (Dai 1999). The two new species of *Aparapotamon* collected from Yunnan Province are herein described. Morphological comparisons were made between the two new species and type materials of other eleven species of *Aparapotamon*. To analyze the phylogenetic relationship between these species and its congeners, we use the mitochondrial 16S rRNA gene for phylogenetic analysis, which has been proved to be useful in crab taxonomy (Schubart 2000; Bai et al. 2018).

## Materials and methods

Specimens were collected by Han Dai from Biji Village (25°53'34"N, 100°55'30"E, alt. 1658 m), Lawu Town, Binchuan County, Dali Bai Autonomous Prefecture, Yunnan Province and Yue Huang from Zebu Village (26°30'41"N, 103°10'25"E, alt. 1954 m), Nagu Town, Huize County, Qujing City, Yunnan Province, respectively. All materials were preserved in 95% ethanol and deposited in the Department of Parasitology of the Medical College of Nanchang University, Jiangxi, China (**NCU MCP**). Carapace width and length were measured in millimeters. The abbreviation of G1 and G2 are for male first gonopod and the male second gonopod, respectively. The terminology used primarily follows that of Dai (1999) and Davie et al. (2015).

We compared two new species with type materials of other eleven species of *Aparapotamon* deposited in Chinese Academy of Sciences, Beijing, China (CAS CB). Comparative materials are as follows:

- Aparapotamon arcuatum Dai & Chen, 1985: Holotype, CAS CB 05091, 1♂, China, Yunnan Province, Lijiang City, Ninglang Yi Autonomous County, Daxing Town, 14 Aug 1981; NCU MCP 4032, 1♂, China, Yunnan Province, Lijiang City, Yongsheng County, Yangping Yi Autonomous Town, 6 Jul 2017.
- Aparapotamon emineoforaminum Dai & Chen, 1985: Holotype, CAS CB 05090, 1♂, China, Sichuan Province, Liangshan Yi Autonomous Prefecture, Mianning County, Jionglong Town, Aug 1982.
- Aparapotamon gracilipedum Chen & Chang, 1982: Holotype, CAS CB 05148, 1♂, China, Henan Province, Luoyang City, Luanchuan County, Chenguan Town, 20 Sep 1978.
- Aparapotamon grahami Rathbun, 1929: CAS CB 00142, 1♂, China, Hubei Province, Nanyang City, 1977; CAS CB 00150, 1♂, China, Shannxi Province, Ankang City, Zhenping County, 16 Jul 1978; NCU MCP 4057, 1♂, China, Chongqing City, Wulong County, Dadonghe Town, 24 Jun 2018; NCU MCP 4241, 1♂, China, Yunnan Province, Kunming City, 31 Aug 2019.
- Aparapotamon huiliense Dai & Chen, 1985: Holotype, CAS CB 05089, 1♂, China, Sichuan Province, Liangshan Yi Autonomous Prefecture, Huili County, 2 Jun 1982; NCU MCP 4027, 1♂, China, Yunnan Province, Lijiang City, Huaping County, Zhongxin Town, Zuofang Village, 5 Jul 2017.
- Aparapotamon inflomanum Dai & Chen, 1985: Holotype, CAS CB 05096, 1♂, China, Yunnan Province, Diqing Zang Autonomous Prefecture, Zhongdian County, Sanba Town, 8 Sep 1981.
- Aparapotamon molarum Dai & Chen, 1985: Holotype, CAS CB 05094, 1♂, China,Yunnan Province, Lijiang City, Yulong Naxi Autonomous County, Jade Dragon Snow Mountain, 28 Aug 1981.
- Aparapotamon muliense Dai & Chen, 1990: Holotype, CAS CB 05088, 1♂, China, Sichuan Province, Liangshan Yi Autonomous Prefecture, Muli Zang Autonomous County, Xiaojin River, 5 Dec 1984.
- Aparapotamon protinum Dai & Chen, 1985: Holotype, CAS CB 05093, 1♂, China, Yunnan Province, Lijiang City, Yongsheng County, Songping Town, 22 Aug 1981.
- Aparapotamon similium Dai & Chen, 1985: Holotype, CAS CB 05095, 1♂, China, Yunnan Province, Lijiang City, Yongsheng County, Renli Town, 22 Aug 1981; NCU MCP 4031, 1♂, China,Yunnan Province, Lijiang City, Ninglang Yi Autonomous County, Paomaping Town, 6 Jul 2017.
- Aparapotamon tholosum Dai & Chen, 1985: Holotype, CAS CB 05092, 1♂, China, Yunnan Province, Lijiang City, Yongsheng County, Chenguan Town, 22 Aug 1981; NCU MCP 4034, 1♂, China, Yunnan Province, Dali Bai Autonomous Prefecture, Binchuan County, Zhoucheng Town, 5 Jul 2017.

Institutional abbreviations used in the paper are as follows:

**CAS CB**Chinese Academy of Sciences, Beijing, China;

**NCHUZOOL**Zoological Collections of the Department of Life Science, National Chung Hsing University, Taichung, Taiwan;

**NCU MCP**Department of Parasitology of the Medical College of Nanchang University, Jiangxi, China;

**NNU** College of Life Sciences, Nanjing Normal University, Nanjing, China;

**SYSBM** Sun Yat-sen Museum of Biology, Sun Yat-Sen University, Guangzhou, China;

**ZRC**Zoological Reference Collection of the Raffles Museum of Biodiversity Research, National University of Singapore, Singapore.

The pereiopod muscle tissue was extracted from specimens of the new species with a DP1902 Tissue Kit (BioTeKe Inc. Beijing). Partial mitochondrial 16S rRNA gene sequences were obtained by PCR amplification with the primers 1471 (5’-CCTGTTTANCAAAAACAT-3’) and 1472 (5’-AGATAGAAACCAACCTGG-3’) (Shih et al. 2004). The parameters of the PCR were as follows: denaturation for 50 s at 94 °C, annealing for 40 s at 52 °C, extension for 1 min at 72 °C (33 cycles) and extension for 10 min at 72 °C. The PCR products were examined on an ABI 3730 automatic sequencer to sequence.

For molecular analysis, 30 partial sequences of 16S rRNA gene were used to construct BI and ML phylogenetic trees, including those of 27 species in 22 genera of potamids (Table 1). Sequences were aligned using MAFFT vers.7.355 (Nakamura et al. 2018) based on the G-INS-I method and the conserved regions were selected with Gblocks 0.91b (Castresana 2000). The best-fitting model for Bayesian Inference (BI) analysis was determined by MrModeltest ver. 2.3 (Nylander 2004), selected by the Akaike information criterion (AIC). The obtained model was GTR+I+G. MrBayes 3.2.6 (Ronquist et al. 2012) was employed to perform BI analysis, and four Monte Carlo Markov Chains of 2,000,000 generations were run with sampling every 1,000 generations. The first 500,000 generations were discarded as burn-in. The best evolutionary model for Maximum Likelihood (ML) analysis was HKY+G, determined by MEGA X (Kumar et al. 2018) based on the Bayesian information criterion (BIC). A ML tree was built based on 1000 bootstrap replicates in MEGA X (Kumar et al. 2018). The pairwise distance based on the K2P (Kimura 2-Parameter) model was calculated by MEGA X (Kumar et al. 2018).

**Table 1.** Specimens used in the phylogenetic analysis.

Species	Museum catalogue no.	Locality	GenBank no.	Reference
* Aparapotamon grahami *	ZRC	Yunnan, China	AB428489	Shih et al. 2009
*Cryptopotamonanacoluthon* Kemp, 1918	NCHUZOOL 13122	Hong Kong	AB428453	Shih et al. 2009
*Daipotamonminos* Ng & Trontelj, 1996	ZRC	Guizhou, China	LC198524	Huang et al. 2017b
*Diyutamoncereum* Huang, Shih & Ng, 2017	SYSBM	Guizhou, China	LC198520	Huang et al. 2017b
*Mediapotamonleishanense* Dai, 1995	SYSBM001094	Guizhou, China	LC155164	Shih et al. 2016
*Minpotamonnasicum* Dai & Chen, 1979	NCHUZOOL 13121	Fujian, China	AB428450	Shih et al. 2009
*Nanhaipotamonhongkongense* Shen, 1940	ZRC	Hong Kong, China	AB212869	Shih et al. 2005
*Parapotamonspinescens* Calman, 1905	NCU MCP	Yunnan, China	AB428467	Shih et al. 2009
*Pararangunasemilunatum* Dai & Chen, 1985	ZRC	Yunnan, China	AB428490	Shih et al. 2009
*Potamiscusyongshengense* Dai & Chen, 1985	NNU150951	Yunnan, China	KY963597	Chu et al. 2017
*Socotrapotamonnojidensis* Apel & Brandis, 2000	ZRC 2000.2232	Socotra, Yemen	AB428493	Shih et al. 2009
*Tenuipotamonhuaningense* Dai & Bo, 1994	CAS CB05175	Yunnan, China	AB428491	Shih et al. 2009
*Trichopotamondaliense* Dai & Chen, 1985	NCHUZOOL 13130	Yunnan, China	AB428492	Shih et al. 2009
*Yarepotamonfossor* Huang, 2018	SYSBM 001417	Guangxi, China	MG709238	Huang 2018
*Artopotamonlatopeos* Chu, Wang & Sun, 2018	NNU 170502	Yunnan, China	MH045061	Chu et al. 2018b
*Arquatopotamonjizushanense* Chu, Zhou & Sun, 2017	NNU 160506 (holotype)	Yunnan, China	KY963596	Chu et al. 2017
*Semicircularalincangensis* Chu, Wang & Sun, 2018	NNU 1605	Yunnan, China	MH045059	Chu et al. 2018b
*Tenuilapotamonlatilum* Chen, 1980	ZRC	Hubei, China	AB428468	Shih et al. 2009
*Sinopotamondavidi* Rathbun, 1904	CAS CB	Shaanxi, China	LC155132	Shih et al. 2016
*Tiwaripotamonxiurenense* Dai & Naiyanetr, 1994	CAS CB	Guangxi, China	LC198522	Huang et al. 2017b
*Cantopotamonzhuhaiense* Huang, Ahyong & Shih, 2017	SYSBM 001439	Guangdong, China	LC342045	Huang et al. 2017a
*Qianguimonsplendidum* Huang, 2018	SYSBM 001598	Guangxi, China	MG709241	Huang 2018
* Artopotamon compressum *	NCU MCP 4033	Yunnan, China	MN594116	This study
* Aparapotamon huiliense *	NCU MCP 4027	Yunnan, China	MN594113	This study
* Aparapotamon huiliense *	NCU MCP 4031	Yunnan, China	MN594118	This study
* Aparapotamon similium *	NCU MCP 4035	Yunnan, China	MN594114	This study
*Aparapotamonbinchuanense* sp. nov.	NCU MCP 1707	Yunnan, China	MN943639	This study
*Aparapotamonbinchuanense* sp. nov.	NCU MCP 1707	Yunnan, China	MN594120	This study
*Aparapotamonhuizeense* sp. nov.	NCU MCP 1798	Yunnan, China	MN594121	This study
*Aparapotamonhuizeense* sp. nov.	NCU MCP 1798	Yunnan, China	MN594122	This study

## Results

### Systematics

#### Family Potamidae Ortmann, 1896

##### Aparapotamon

Taxon classificationAnimaliaDecapodaPotamidae

Dai & Chen, 1985

2F4642BD-22F8-5749-9BE8-803810E2A1BC

###### Type species.

*Aparapotamongrahami* Dai & Chen, 1985

##### Aparapotamon binchuanense sp. nov.

Taxon classificationAnimaliaDecapodaPotamidae

87CADA95-2FCA-5C9E-847E-824DCA7C6866

http://zoobank.org/05703d3e-5f19-4587-9494-c1afb7df8327

**Figure 1.**
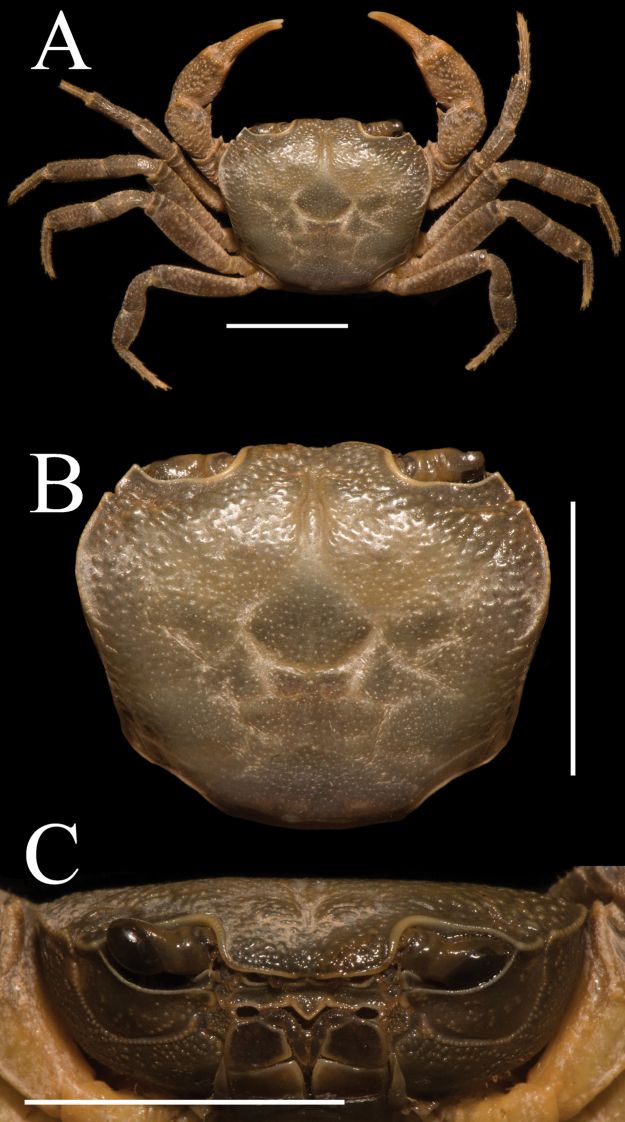
*Aparapotamonbinchuanense* sp. nov. Holotype male (17.1 × 13.6 mm) (NCU MCP 170701). **A** overall habitus **B** dorsal view of carapace **C** frontal view of the cephalothorax. Scale bars: 1 cm.

**Figure 2.**
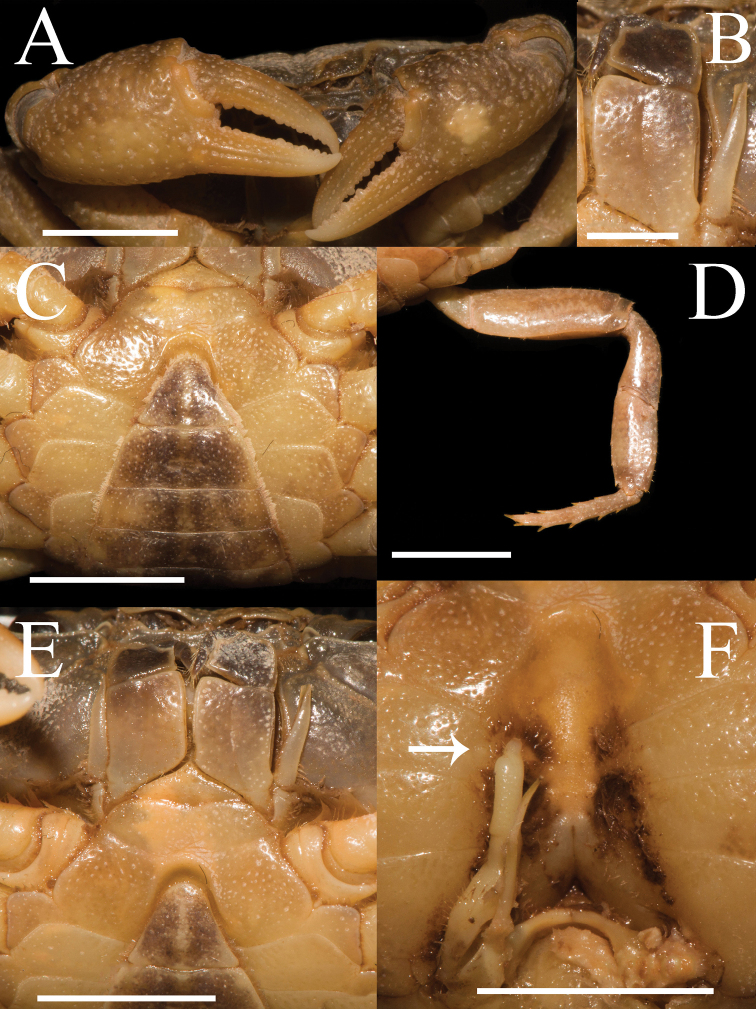
*Aparapotamonbinchuanense* sp. nov. Holotype male (17.1 × 13.6 mm) (NCU MCP 170701). **A** outer view of chelipeds **B** left third maxilliped **C** ventral view of anterior thoracic sternum and pleon **D** right fourth ambulatory leg **E** ventral view of anterior thoracic sternum and third maxilliped **F** ventral view of sterno-pleonal cavity with right G1 in situ; arrow indicates pleonal locking tubercle. Scale bars: 2 mm (**B**); 5 mm (**A, C–F**).

**Figure 3.**
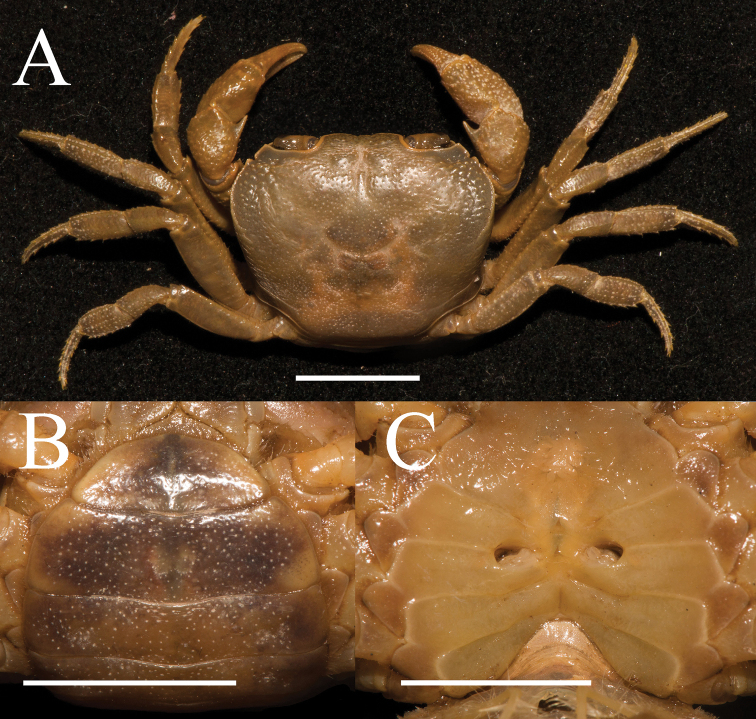
*Aparapotamonbinchuanense* sp. nov. Paratype female (21.4 × 17.1 mm) (NCU MCP 170703) **A** overall habitus **B** ventral view of pleon **C** vulvae. Scale bars: 1 cm.

**Figure 4.**
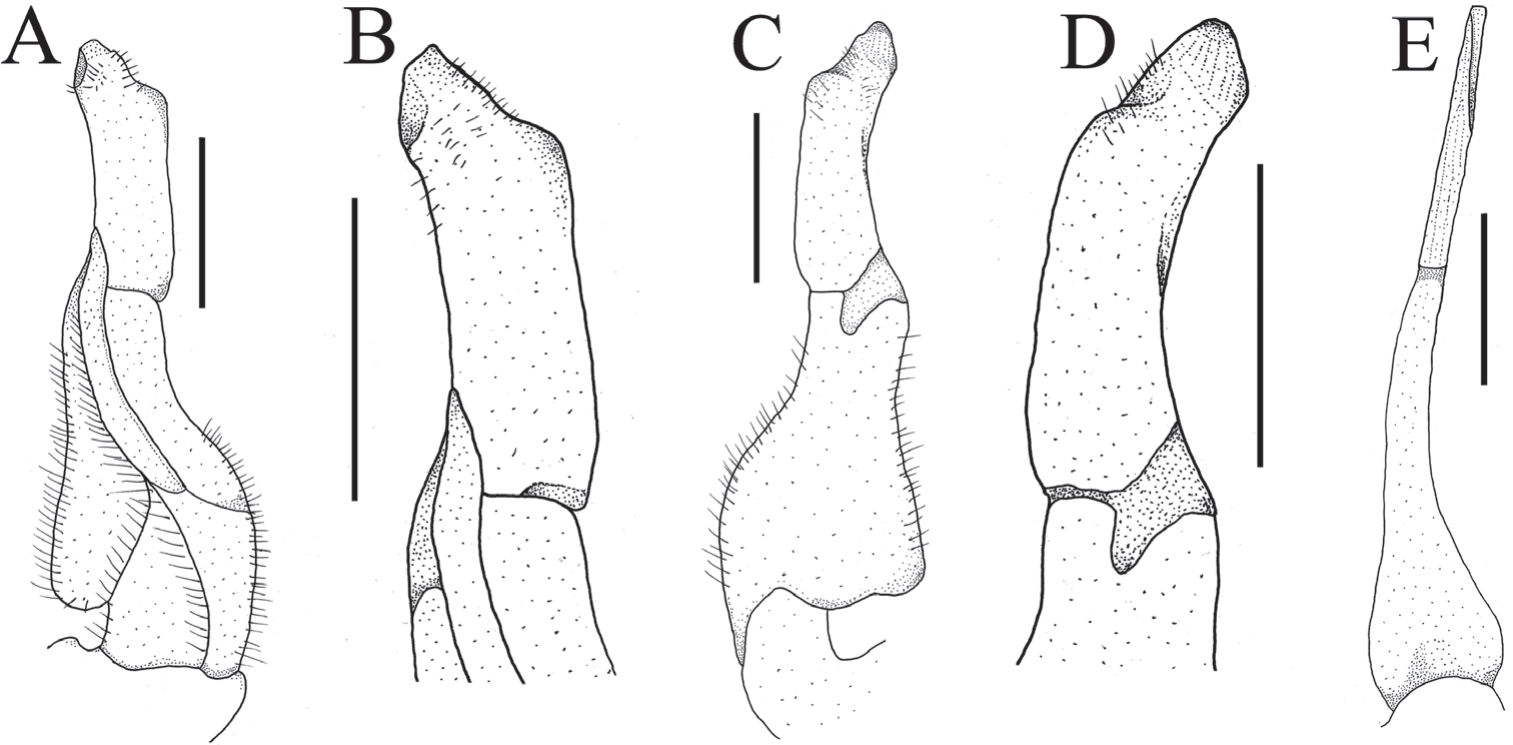
*Aparapotamonbinchuanense* sp. nov. Holotype male (17.1 × 13.6 mm) (NCU MCP 170701) **A** ventral view of left G1**B** ventral view of terminal segment of left G1**C** dorsal view of left G1**D** dorsal view of terminal segment of left G1**E** ventral view of left G2. Scale bars: 1 mm.

Figures 1 , 2 , 3 , 4

###### Material examined.

***Holotype***: NCU MCP 170701, 1♂ (17.1 × 13.6 mm), China, Yunnan Province, Dali Bai Autonomous Prefecture, Binchuan County, Lawu Town, 25°53'34"N, 100°55'30"E, alt. 1658 m, 10 Aug 2010, Han Dai leg. ***Paratypes***: NCU MCP 170702, NCU MCP 170704, NCU MCP 170705, 3♂♂ (15.7 × 13.1 mm, 15.6 × 12.5 mm, 14.3 × 11.6 mm) and NCU MCP 170703, NCU MCP 170706, NCU MCP 170707, 3♀♀ (21.4 × 17.1 mm, 20.8 × 16.8 mm, 19.0 × 15.6 mm), same data as holotype.

###### Diagnosis.

Carapace trapezoidal, regions defined. External orbital angle triangular, postorbital cristae convex, postfrontal lobe prominent. Cervical groove indistinct, H-shaped groove conspicuous. Epibranchial tooth blunt, anterolateral margin lined with numerous granules. Third maxilliped exopod without flagellum. Adult male and female chelipeds slightly unequal. Ambulatory legs relatively slender. Male sterno-pleonal cavity deep, median longitudinal groove between sternites 7/8 long. Male pleon narrow triangular, telson triangular. Vulva small, ovate, located close to each other at anterior part of sternites 6, posterior margin not convex. G1 slender, distal end tapering, distinctly bent. G2 basal segment ovate, tip of terminal segment laterally flattened.

###### Description.

Carapace width 1.25 × length (n = 7), regions defined; dorsal surface slightly convex (Figs 1A, B, 3A). External orbital angle triangular, separated from anterolateral margin by conspicuous notch (Figs 1A, C, 3A). Postorbital cristae gently convex, continuous to epibranchial tooth; postfrontal lobe prominent, separated medially by inverted Y-shaped groove (Figs 1A, B, 3A). Cervical groove indistinct; H-shaped gastro-cardiac groove distinct (Figs 1A, B, 3A). Epibranchial tooth blunt, rounded; anterolateral margin cristae, curved inward posteriorly, lined with approximately 15–17 fused granules; posterolateral surface slightly smooth, with some inconspicuous oblique striae, converging towards posterior carapace margin (Figs 1A, B, 3A). Orbits and eyes large; supraorbital margin ridged, infraorbital margin cristate, minutely granulated (Fig. 1C). Pterygostomial and sub-hepatic regions covered with dense round granules, sub-orbital region with sparse granules (Fig. 1C). Epistome posterior margin median lobe equilateral triangular, lateral margin with small projection (Fig. 1C).

Third maxilliped exopod without flagellum, claviform, reaching proximal 1/3 of merus lateral margin (Figs 1C, 2B, E). Ischium about 1.3 times as long as broad, rectangular, with distinct longitudinal median sulcus (Fig. 2B, E). Merus about 1.4 times as broad as long, subquadrate, median slightly depressed (Figs 1C, 2B, E).

Chelipeds slightly unequal in both adult male and female, right cheliped larger (Fig. 2A). Palm of larger cheliped length 1.4 × height (n = 7); dactylus 0.7 × palm length (n = 7); dactylus as long as pollex (Figs 1A, 2A, 3A). Merus outer surface punctate; carpus surface covered with several prominent granules and sharp spine at inner-distal angle (Figs 1A, 3A). Occlusal margins of fingers of adult male with numerous sparse round blunt teeth, with narrow gap when fingers closed (Fig. 2A). Ambulatory legs very slender; second ambulatory legs longest; fourth ambulatory leg propodus 2.1 × as long as broad (n = 7), shorter than dactylus, which accompanied with several thorn-like spines (n = 7) (Figs 1A, 2D).

Male thoracic sternum punctate, formed by tidy depression; sternites 1–4 broad, sternites 1/2 completely continuous; suture 2/3 complete, transverse; suture 3/4 visible, mesially reaching distolateral part of sterno-pleonal cavity (Fig. 2E). Male sterno-pleonal cavity deep; median longitudinal groove between sternites 7/8 long; male pleonal locking tubercle inconspicuous, positioned medially on sternite 5 (Fig. 2F, arrow). Male pleon narrow triangular (Fig. 2C); telson triangular, apex rounded, width 1.3 × length in males (n = 4), 1.8 × in females (n = 3); somite 6 trapezoidal, width 2.5 × length in males (n = 4), 3.3 × in females (n = 3) (Figs 2C, 3B). Vulvae small, ovate, located close to each other at anterior part of sternites 6, pushing mesial portions of sutures 5/6 forward, deeper laterally, posterior margin not convex, the sternal vulvar cover triangular, positioned mesially (Fig. 3C).

G1 slender; terminal segment claviform, distal end tapering, distinctly bent, inner margin arc-shaped, outer margin straight, dorsal lobe barely visible in ventral view (Fig. 4A–D); tip reaching beyond pleonal locking tubercle but not exceed sternites 4/5 in situ (Fig. 2F); clear boundary between terminal segment and subterminal segment, latter length about 0.7 × length of terminal segment (Fig. 4A, C). G2 basal segment ovate, about 1.5 × length of terminal segment, tip of terminal segment flat rather than sharp (Fig. 4E).

###### Etymology.

The species is named after the type locality, Binchuan County, Dali Bai Autonomous Prefecture, Yunnan Province.

###### Distribution.

The new species is presently known only from the type locality, Binchuan County, Dali Bai Autonomous Prefecture, Yunnan Province.

###### Remarks.

*Aparapotamonbinchuanense* sp. nov. closely resembles congeners in general carapace morphology. However, *A.binchuanense* sp. nov. can be distinguished from other species by the terminal segment of G1, which is claviform, with distal end tapering and distinctly bent (Fig. 9A) [vs. terminal segment of G1 disc-shaped, straight in *A.inflomanum* and *A.molarum* (Fig. 9C, D), terminal segment of G1 of *A.emineoforaminum* tapering distally but not bent (Fig. 9E), terminal segment of G1 arc-shaped in *A.arcuatum* and *A.muliense* (Fig. 9H, I), and terminal segment of G1 of *A.tholosum*, *A.protinum*, *A.grahami*, *A.huiliense*, *A.similium* and *A.gracilipedum* claviform, not bent (Fig. 9F, G, J-M)]. In addition, in *A.binchuanense* sp. nov., the pterygostomial region is densely covered with round granules, while in the sub-orbital region the granules are sparse.

(Fig. 1C). This character can also distinguish *A.binchuanense* sp. nov. from congeners. For detailed differences between this new species and congeners, see Table 2.

##### Aparapotamon huizeense sp. nov.

Taxon classificationAnimaliaDecapodaPotamidae

C5D7F5FB-EA18-5276-886B-2EABADE20A52

http://zoobank.org/9b44a1c4-162b-4db0-be6c-dce5124412b0

**Figure 5.**
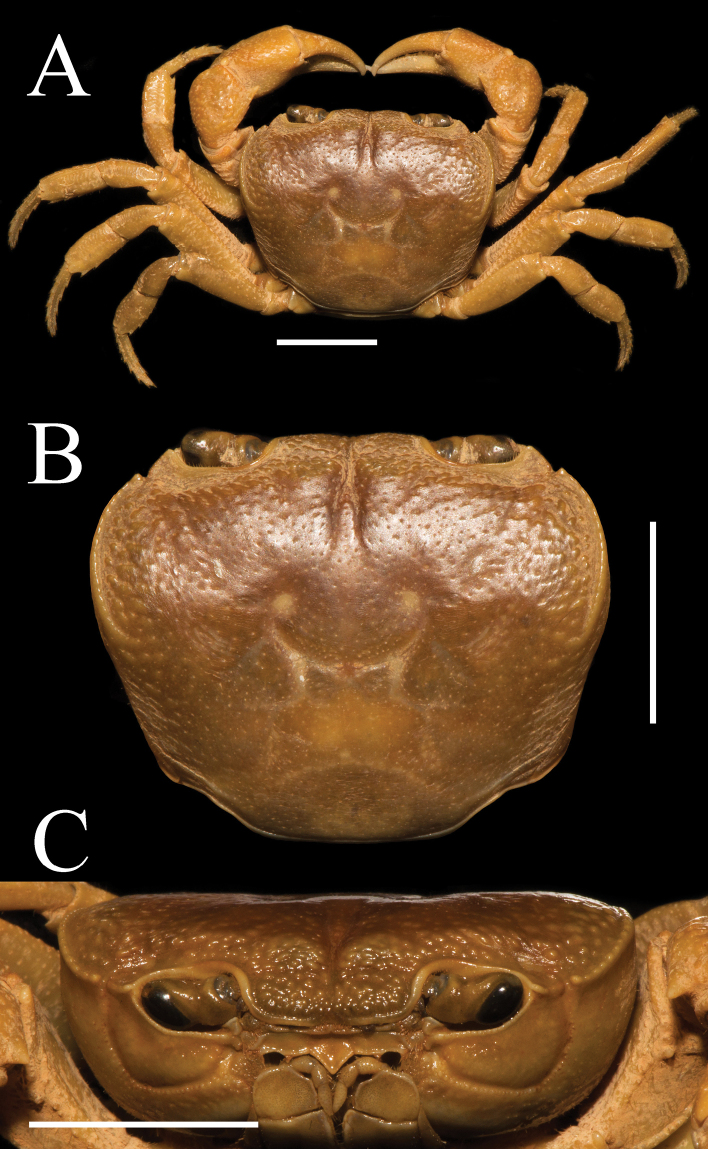
*Aparapotamonhuizeense* sp. nov. Holotype male (25.9 × 21.2 mm) (NCU MCP 179801) **A** overall habitus **B** dorsal view of carapace **C** frontal view of cephalothorax. Scale bars: 1 cm.

**Figure 6.**
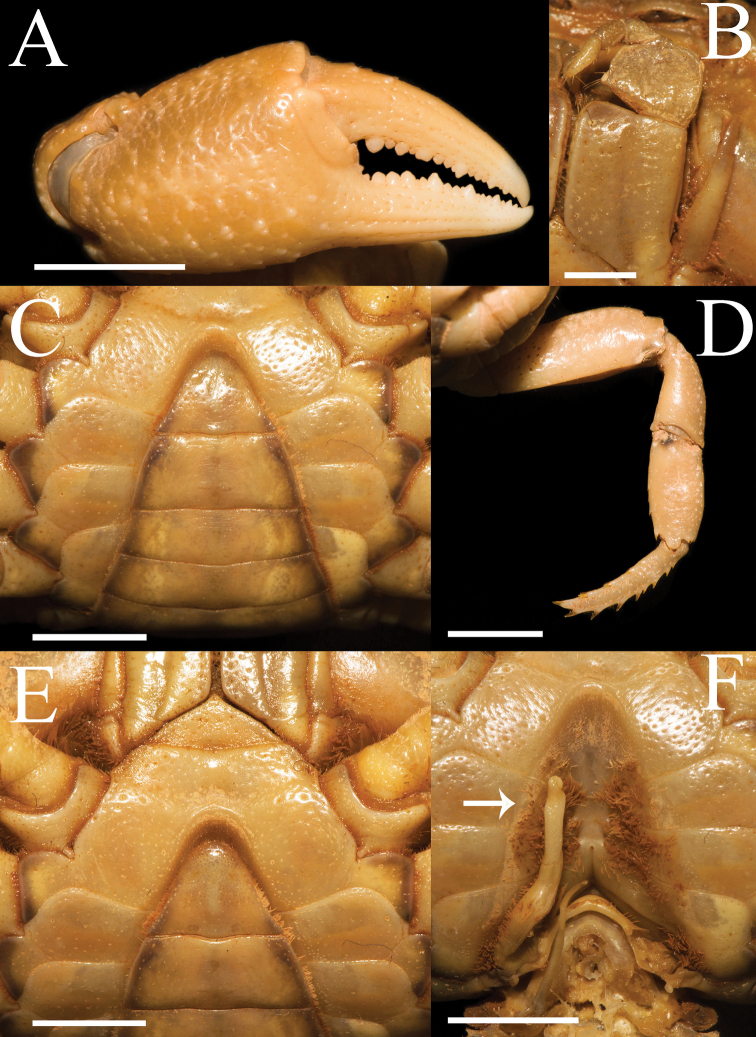
*Aparapotamonhuizeense* sp. nov. Holotype male (25.9 × 21.2 mm) (NCU MCP 179801) **A** outer view of right cheliped **B** left third maxilliped **C** ventral view of pleon **D** right fourth ambulatory leg **E** ventral view of anterior thoracic sternum and telson **F** ventral view of sterno-pleonal cavity with right G1 in situ; arrow indicates pleonal locking tubercle. Scale bars: 2 mm (**B**); 5 mm (**A, C–F**).

**Figure 7.**
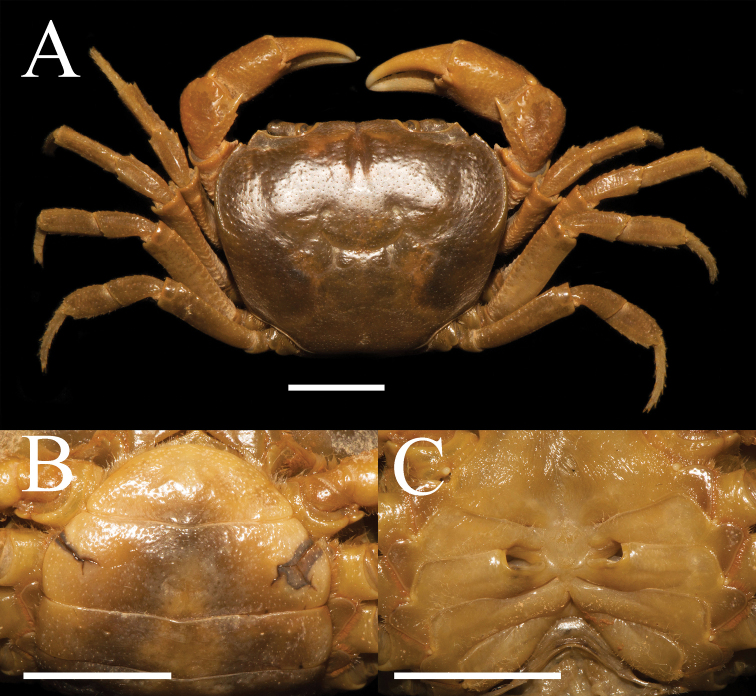
*Aparapotamonhuizeense* sp. nov. Paratype female (31.0 × 24.8 mm) (NCU MCP 179803) **A** overall habitus **B** ventral view of pleon **C** vulvae. Scale bars: 10 mm.

**Figure 8.**
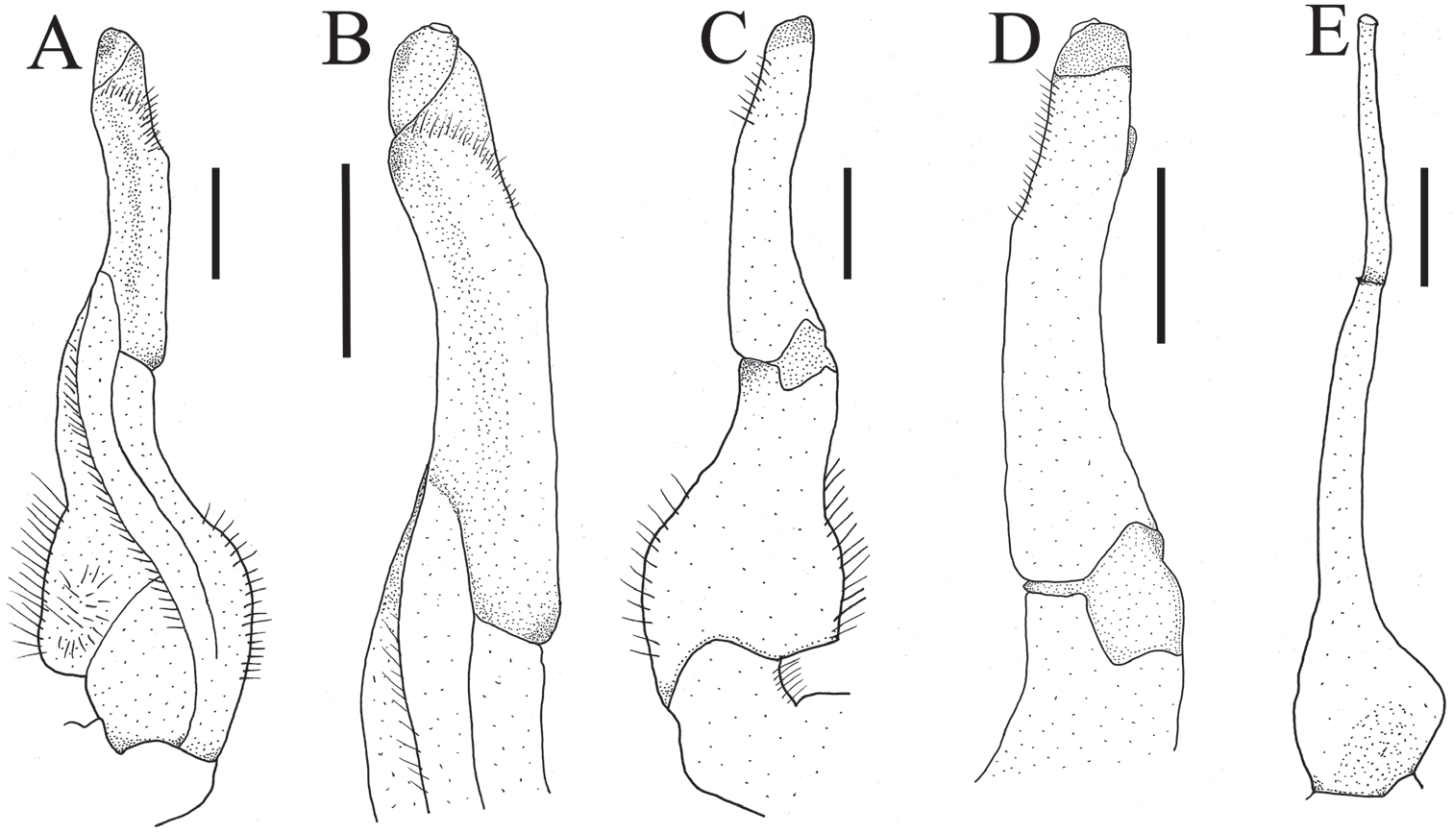
*Aparapotamonhuizeense* sp. nov. Holotype male (25.9 × 21.2 mm) (NCU MCP 179801) **A** ventral view of left G1**B** ventral view of terminal segment of left G1**C** dorsal view of left G1**D** dorsal view of terminal segment of left G1**E** ventral view of left G2. Scale bars: 1 mm.

Figures 5 , 6 , 7 , 8

###### Material examined.

***Holotype***: NCU MCP 179801, 1♂ (25.9 × 21.2 mm), China, Yunnan Province, Qujing City, Huize County, Nagu Town, Zebu Village, 26°30'41"N, 103°10'25"E, alt. 1954 m, 25 Aug 2011, Yue Huang leg. ***Paratypes***: NCU MCP 179802, 1♂ (26.9 × 21.9 mm) and NCU MCP 179803–179808, 6♀♀ (31.0 × 24.8 mm, 30.7 × 23.6 mm, 27.3 × 21.5 mm, 23.5 × 18.4 mm, 25.5 × 20.5 mm, 29.8 × 22.6 mm), same data as holotype.

###### Diagnosis.

Carapace trapezoidal, dorsal surface slightly convex, regions defined. External orbital angle round, separated from anterolateral margin, postorbital cristae convex, postfrontal lobe prominent. Cervical groove shallow, H-shaped groove distinct, especially in female specimen. Epibranchial tooth distinct, especially in female specimen. Third maxilliped exopod without flagellum. Ambulatory legs slender. Male pleon broad triangular, telson triangular, apex rounded. Vulva ovate, covering anterior half of sternite 6, with the posterior margin distinctly convex. G1 very slender, dorsal lobe well developed, exceeding suture 4/5 in situ, G2 basal segment ovate, tip of terminal segment round.

###### Description.

Carapace width 1.25 × length (n = 8), regions distinctly defined; dorsal surface slightly convex, anterolateral and frontal region covered with conspicuous round granules (Fig. 5A, B). External orbital angle triangular, round, separated from anterolateral margin by deep notch (Figs 5A–C, 7A). Postorbital cristae convex, not continuous to epibranchial tooth; postfrontal lobe prominent, separated medially by a Y-shaped groove extending to the frontal region (Figs 5A, B, 7A). Cervical groove shallow; H-shaped gastro-cardiac groove distinct, especially in female specimen (Figs 5A, B, 7A). Epibranchial tooth sharp, distinct, especially in female specimen; anterolateral margin cristae distinct, curved inwards posteriorly, lined with approximately 10–13 ambiguous granules; posterolateral surface smooth, with some inconspicuous oblique striae, converging towards posterior carapace margin (Figs 5A, B, 7A). Orbits and eyes medium-size; supraorbital margin ridged, infraorbital margins cristate, minutely granulated (Fig. 5C). Sub-orbital smooth, pterygostomial and sub-hepatic regions covered with sparse round granules (Fig. 5C). Epistome posterior margin median lobe broad triangular, lateral margin with small projection (Fig. 5C).

Third maxilliped exopod without flagellum, claviform, reaching proximal 1/3 of merus lateral margin (Figs 5C, 6B). Ischium about 1.4 times as long as broad, rectangular, longitudinal median sulcus indistinct (Fig. 6B). Merus about 1.3 times as broad as long, subquadrate, median slightly depressed (Figs 5C, 6B). Chelipeds unequal in both adult male and female, palm of larger cheliped length 1.4 × height (n = 8); dactylus 0.6 × palm length (n = 8); slightly shorter than pollex (Figs 5A, 6A). Merus outer surface punctate; carpus surface covered with several prominent granules and sharp spine at inner-distal angle (Figs 5A, 7A). Occlusal margins of fingers of adult male with numerous round blunt teeth, with narrow gap when fingers closed (Fig. 6A). Ambulatory legs slender; second ambulatory legs longest; fourth ambulatory leg propodus 1.9 × as long as broad (n = 8), shorter than dactylus, which accompanied with several thorn-like setae (Figs 5A, 6D).

Male thoracic sternum punctate, formed by tidy depression; sternites 1–4 broad, sternites 1/2 completely continuous; suture 2/3 complete, transverse; suture 3/4 visible, mesially reaching distolateral part of sterno-pleonal cavity (Fig. 6C, E). Male sterno-pleonal cavity deep; median longitudinal groove between sternites 7, 8 long; male pleonal locking tubercle barely visible, almost middle of sternite 5 (Fig. 6F, arrow). Male pleon broad triangular (Fig. 6C); telson triangular, apex rounded, width 1.4 × length in males (n = 2), 2.5 × in females (n = 6); somite 6 trapezoidal, width 2.3 × length in males (n = 2), 3.0 × in females (n = 6) (Figs 6C, 7B). Vulva medium-size, ovate, superior margin reaching suture 5/6 in situ, opening inward, posterior margin distinctly convex, the sternal vulvar cover broadly triangular and relatively low (Fig. 7C).

G1 very slender; terminal segment claviform, slightly bent distally, inner margin arc-shaped, outer margin straightly, dorsal lobe well developed and gonopod pore located in it (Fig. 8A–D); exceeding suture4/5 in situ (Fig. 6F); clear boundary between terminal segment and subterminal segment, the latter length about 0.9 × length of terminal segment (Fig. 8A, C). G2 basal segment ovate, about 1.9 × length of terminal segment, tip of terminal segment round (Fig. 8E).

###### Etymology.

The species is named after the type locality, Huize County, Qujing City, Yunnan Province.

###### Distribution.

The new species is presently known only from the type locality presently, Huize County, Qujing City, Yunnan Province.

###### Remarks.

**Figure 9.**
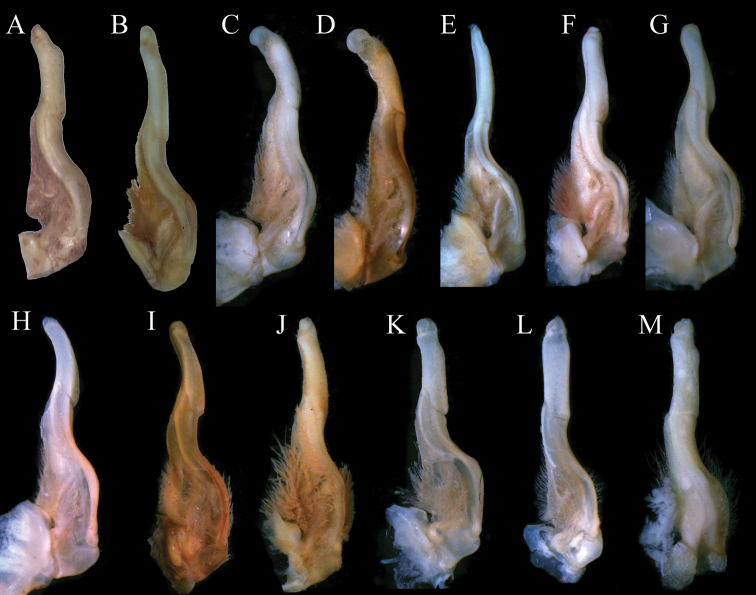
Left G1s. **A***Aparapotamonbinchuanense* sp. nov. NCU MCP 170701 **B***Aparapotamonhuizeense* sp. nov. NCU MCP 179801 **C***Aparapotamoninflomanum* (Dai & Chen, 1985), IZCAS CB 05096 **D***Aparapotamonmolarum* (Dai & Chen, 1985), CAS CB 05094 **E***Aparapotamonemineoforaminum* (Dai & Chen, 1985), CAS CB 05090 **F***Aparapotamontholosum* (Dai & Chen, 1985), CAS CB 05092 **G***Aparapotamonprotinum* (Dai & Chen, 1985), CAS CB 05093 **H***Aparapotamonarcuatum* (Dai & Chen, 1985), CAS CB 05091 **I***Aparapotamonmuliense* (Dai & Chen, 1990), CAS CB 05088 **J***Aparapotamongrahami* (Rathbun, 1929), CAS CB 00142 **K***Aparapotamonhuiliense* (Dai & Chen, 1985), CAS CB 05089 **L***Aparapotamonsimilium* (Dai & Chen, 1985), CAS CB 05095 **M***Aparapotamongracilipedum* (Chen & Chang, 1982), CAS CB 05148.

*Aparapotamonhuizeense* sp. nov. closely resembles *A.grahami* in the general carapace morphology and G1 structure. However, *A.huizeense* sp. nov. can be distinguished from *A.grahami* by the following characters: G1 exceeding suture 4/5 in situ (Fig. 6F) [vs. reaching pleonal locking tubercle but not reaching suture 4/5 in situ (Dai 1999: fig. 187)]; and the G1 is very slender, terminal segment slightly bent distally, dorsal lobe well developed (Fig. 9B) [vs. slender, terminal segment without bending (Fig. 9J), dorsal lobe variably developed]. *A.huizeense* sp. nov. is also similar to *A.huiliense*. But, in *A.huiliense*, G1 extends to pleonal locking tubercle but not exceeding suture 4/5 in situ (Dai 1999: fig. 189) and its dorsal lobe roundly developed (Fig. 9K). For detailed differences between this new species and congeners, see Table 2.

**Table 2.** Morphological differences among species of *Aparapotamon*.

Species/characters	Epibranchial tooth	Pterygostomial and sub-hepatic regions	Sub-orbital region	G1 in situ	Terminal segment of G1	Vulva
*A.binchuanense* sp. nov.	Blunt (Fig. 1A)	Densely covered with round granules (Fig. 1C)	Sparely covered with round granules (Fig. 1C)	Exceeding pleonal locking tubercle but not suture 4/5 (Fig. 2F)	Slender, distal end tapering, distinctly bent (Fig. 9A)	Ovate, posterior margin not convex (Fig. 3C)
*A.huizeense* sp. nov.	Sharp (Fig. 5A)	Sparely covered with round granules (Fig. 5C)	Smooth (Fig. 5C)	Exceeding suture 4/5 (Fig. 6F)	Very slender, distal end slightly bent, dorsal lobe well developed inward (Fig. 9B)	Ovate, posterior margin distinctly convex (Fig. 7C)
*A.inflomanum* (cf. Dai 1999: fig. 196)	Blunt	Smooth	Smooth	Reaching suture 4/5	Slender, distal end disc-shaped (Fig. 9C)	Ovate, posterior margin not convex
*A.molarum* (cf. Dai 1999: fig. 195)	Blunt	Smooth	Smooth	Exceeding suture 4/5	Slender, distal end disc-shaped (Fig. 9D)	Transversely ovate, posterior margin not convex
*A.emineoforaminum* (cf. Dai 1999: fig. 197)	Blunt	Densely covered with round granules	Smooth	Exceeding suture 4/5	Very slender, tapering distally (Fig. 9E)	Ovate, posterior margin distinctly convex
*A.tholosum* (cf. Dai 1999: fig. 194)	Sharp	Densely covered with round granules	Smooth	Exceeding pleonal locking tubercle but not suture 4/5	Slender, dorsal lobe well developed upwards (Fig. 9F)	Transversely ovate, posterior margin distinctly convex
*A.protinum* (cf. Dai 1999: fig. 193)	Sharp	Densely covered with round granules	Smooth	Exceeding pleonal locking tubercle but not suture 4/5	Slender, dorsal lobe slightly developed upwards (Fig. 9G)	Transversely ovate, posterior margin arching to form semicircular structure
*A.arcuatum* (cf. Dai 1999: fig. 191)	Blunt	Sparely covered with round granules	Smooth	Exceeding pleonal locking tubercle but not suture 4/5	Slender, arc-shaped, dorsal lobe slightly developed upwards (Fig. 9H)	Transversely ovate, posterior margin not convex
*A.muliense* (cf. Dai 1999: fig. 192)	Blunt	Sparely covered with round granules	Smooth	Exceeding pleonal locking tubercle but not suture 4/5	Slender, arc-shaped, dorsal lobe well developed upwards (Fig. 9I)	Transversely ovate, posterior margin distinctly convex
*A.grahami* (cf. Dai 1999: fig. 187)	Sharp	Sparely covered with round granules	Smooth	Reaching pleonal locking tubercle	Slender, dorsal lobe variably developed inwards (Fig. 9J)	Ovate, posterior margin slightly convex
*A.huiliense* (cf. Dai 1999: fig. 189)	Sharp	Sparely covered with round granules	Smooth	Exceeding pleonal locking tubercle but not suture 4/5	Slender, dorsal lobe roundly developed (Fig. 9K)	Transversely ovate, posterior margin slightly convex
*A.similium* (cf. Dai 1999: fig. 188)	Blunt	Densely covered with round granules	Sparely covered with round granules	Exceeding pleonal locking tubercle but not suture 4/5	Slender, dorsal lobe slightly developed inwards, tapering distally (Fig. 9L)	Transversely ovate, posterior margin distinctly convex
*A.gracilipedum* (cf. Dai 1999: fig. 190)	Sharp	Densely covered with round granules	Sparely covered with round granules	Exceeding pleonal locking tubercle but not suture 4/5	Slender, dorsal lobe slightly developed inwards, distal end blunt (Fig. 9M)	Ovate, posterior margin slightly convex

### Phylogenetic analyses

**Figure 10.**
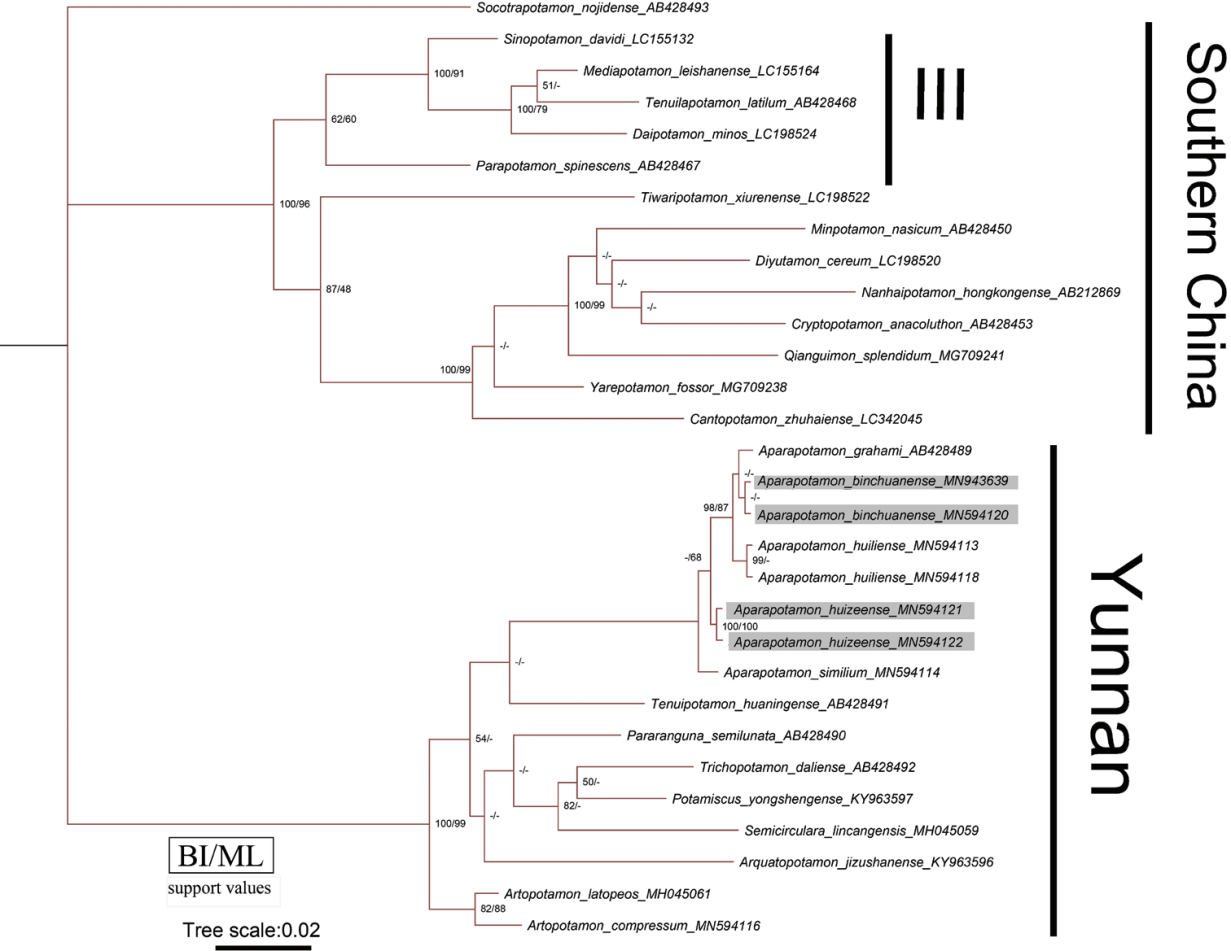
Bayesian inference (BI) phylogenetic tree based on 16S rRNA gene for two new species with their sequence accession numbers see Table 1. Probability values at nodes represent support values for BI and ML. Only values > 50% are shown.

Thirty 529 bp 16S rRNA gene sequences were used to construct BI and ML trees. The phylogenetic tree in this study included five species of *Aparapotamon*, and the results showed that they were clustered into one clade (Fig. 10). Eight sequences of five species were clustered into one branch, including the two new species reported in this paper, along with *A.huiliense*, *A.similium*, and *A.grahami*. The pairwise distance based on the K2P model showed that the pairwise genetic distances among five species of *Aparapotamon* range from 0.0019 to 0.0095 (Table 3). The minimum genetic distance is between *A.grahami* and *A.binchuanense* sp. nov., indicating that they have a close relationship. The minimum genetic distances between *A.huizeense* sp. nov. and other four species is 0.0038, which is same as the genetic distance between *A.grahami* and *A.huiliense*.

**Table 3.** The pairwise genetic distances among five species from *Aparapotamon*.

**species**	**1**	**2**	**3**	**4**	**5**	**6**	**7**	**8**
* A. grahami * AB428489								
* A. similium * MN594114	0.0095							
* A. huiliense * MN594113	0.0038	0.0095						
* A. huiliense * MN594118	0.0038	0.0095	0.0000					
*A.binchuanense* sp. nov. MN943639	0.0019	0.0076	0.0019	0.0019				
*A.binchuanense* sp. nov. MN594120	0.0019	0.0076	0.0019	0.0019	0.0000			
*A.huizeense* sp. nov. MN594121	0.0057	0.0038	0.0057	0.0057	0.0038	0.0038		
*A.huizeense* sp. nov. MN594122	0.0057	0.0038	0.0057	0.0057	0.0038	0.0038	0.0000	

## Discussion

There are currently 13 species in this genus including those described in this study. The original eleven species of *Aparapotamon* are morphologically diverse, with the distal end of G1s of *A.inflomanum* and *A.molarum* being disc-shaped but that of *A.emineoforaminum* tapering distally, and the three G1s extend to suture 4/5, while the other eight species have G1s that are claviform in terminal segment and distal ends do not extend to suture 4/5 (Dai 1999). *A.binchuanense* sp. nov. and *A.huizeense* sp. nov. can be distinguished from above eleven species by their G1s, which distinctly bent in distal end and very slender, dorsal lobe distinctly developed, distal end exceeds suture 4/5 respectively.

In this study, 30 sequences of 16S rRNA gene from 27 species of 22 genera were used to performed phylogenetic analyses. Since the two new species cluster with other *Aparapotamon* species form a separate branch in clade (Fig. 10), the phylogenetic tree supports the assignment of these two new species to *Aparapotamon*. However, the genetic distances between species of *Aparapotamon* are small, ranging from 0.0019 to 0.0095 (Table 3). *A.grahami* and *A.binchuanense* sp. nov. are close at molecular level, with the genetic distance 0.0019, but they are significantly different in morphology (Table 2). Most importantly, *A.binchuanense* sp. nov. can be distinguished from *A.grahami* by the terminal segment of G1, which is claviform, with distal end tapering and distinctly bent (Fig. 9A) [vs. terminal segment of G1*A.grahami* claviform, not bent (Fig. 9J)]. In this study, the molecular results of 16S rRNA gene were not sufficient for species identification in *Aparapotamon*. Therefore, it is recommended to use other markers (e.g. COI and nuclear genes) for further phylogenetic studies of this genus. If the results of other makers indicate that the genetic distance between *Aparapotamon* is also too small compared to other freshwater crab genera, revision of *Aparapotamon* is necessary.

The present molecular results show five species of *Aparapotamon* were clustered into one clade. And *Aparapotamon* cluster with other genera from Yunnan form ‘Yunnan’ clade. The genera in the branch of ‘Yunnan’ have many similarities in terms of morphological structure, such as the G1 slender, the terminal segment is longer than the half of subterminal segment, third maxilliped exopod without flagellum, and the ability to live at an altitude of 1500–2900 meters (Dai and Chen 1985; Dai 1999; Chu et al. 2017). Specimens of *A.molarum* were collected at Baishui River, Yulong Naxi Autonomous County, Lijiang City, Yunnan Province at an altitude of 2910 meters, which is the highest altitude at which freshwater crab specimens have been discovered in China so far (Dai 1999).

## Supplementary Material

XML Treatment for Aparapotamon

XML Treatment for Aparapotamon binchuanense

XML Treatment for Aparapotamon huizeense

## Supplementary materials

Supplementary material 1 BI tree Data type Tre. file Explanation note Phylogenetic. https://binary.pensoft.net/file/579862This dataset is made available under the Open Database License (http://opendatacommons.org/licenses/odbl/1.0/). The Open Database License (ODbL) is a license agreement intended to allow users to freely share, modify, and use this Dataset while maintaining this same freedom for others, provided that the original source and author(s) are credited.Qi-Hong Tan, Xiao-Juan Zhou, Jie-Xin Zou

Supplementary material 2 ML tree Data type MTSX file Explanation note Phylogenetic. https://binary.pensoft.net/file/579863This dataset is made available under the Open Database License (http://opendatacommons.org/licenses/odbl/1.0/). The Open Database License (ODbL) is a license agreement intended to allow users to freely share, modify, and use this Dataset while maintaining this same freedom for others, provided that the original source and author(s) are credited.Qi-Hong Tan, Xiao-Juan Zhou, Jie-Xin Zou

Supplementary material 3 Sequences Data type FASTA. file Explanation note Genomic. https://binary.pensoft.net/file/579864This dataset is made available under the Open Database License (http://opendatacommons.org/licenses/odbl/1.0/). The Open Database License (ODbL) is a license agreement intended to allow users to freely share, modify, and use this Dataset while maintaining this same freedom for others, provided that the original source and author(s) are credited.Qi-Hong Tan, Xiao-Juan Zhou, Jie-Xin Zou
